# Role of NADPH Oxidase in Metabolic Disease-Related Renal Injury: An Update

**DOI:** 10.1155/2016/7813072

**Published:** 2016-08-15

**Authors:** Cheng Wan, Hua Su, Chun Zhang

**Affiliations:** Department of Nephrology, Union Hospital, Tongji Medical College, Huazhong University of Science and Technology, Wuhan, Hubei 430022, China

## Abstract

Metabolic syndrome has been linked to an increased risk of chronic kidney disease. The underlying pathogenesis of metabolic disease-related renal injury remains obscure. Accumulating evidence has shown that NADPH oxidase is a major source of intrarenal oxidative stress and is upregulated by metabolic factors leading to overproduction of ROS in podocytes, endothelial cells, and mesangial cells in glomeruli, which is closely associated with the initiation and progression of glomerular diseases. This review focuses on the role of NADPH oxidase-induced oxidative stress in the pathogenesis of metabolic disease-related renal injury. Understanding of the mechanism may help find potential therapeutic strategies.

## 1. Introduction

Metabolic syndrome is a constellation of interconnected risk factors for cardiovascular diseases and type 2 diabetes, including dyslipidemia, hypertension, hyperglycemia, abdominal obesity, and insulin resistance [1, 2]. Along with cardiovascular diseases and type 2 diabetes, accumulating evidence shows that metabolic syndrome contributes to an increased risk of microalbuminuria and/or chronic kidney disease (CKD) [3–7]. However, it remains unclear whether there is a definitive cause-and-effect relationship between metabolic syndrome and renal injury.

Research on the underlying pathogenesis of metabolic disease-related renal injury has suggested an important role of oxidative stress, which is a result of reactive oxygen species (ROS) overproduction, mitochondrial dysfunction, and/or impaired antioxidant system [8]. There are numerous intrarenal sources of ROS, such as mitochondrial electron transport chain, xanthine oxidase, and uncoupled nitric oxide (NO) synthase, while nicotinamide adenine dinucleotide phosphate (NADPH) oxidase is generally accepted as the major producer [9–13].

NADPH oxidases are multisubunit enzymes composing membrane and cytosolic components that transfer electrons across biological membranes. There are seven members in the Nox family of NADPH oxidase, including Nox1–Nox5 and dual oxidases, Duox1 and Duox2, with different activation mechanisms and tissue distribution [13–16]. The Nox homologues are widely expressed throughout the kidney. Nox1, Nox2, Nox4, and Nox5 are predominantly expressed in glomerular endothelial cells, tubulointerstitial cells, and glomerular cells, that is, mesangial cells and glomerular epithelial cells [17]. Various homologue-specific mechanisms regulate the activity of the Nox family involving a complex series of protein/protein interactions, phosphorylation and translocation of its subunits, and Rac activation. Numerous stimuli and agonists like transforming growth factor-*β* (TGF-*β*), angiotensin II (Ang II), hyperglycemia, oxidized low density lipoprotein (oxLDL), insulin-like growth factor-1 (IGF-1), vascular endothelial growth factor (VEGF), and aldosterone are capable of upregulating the activity and/or the expression of NADPH oxidases, subsequently leading to overproduction of ROS including the immediate product superoxide and the following hydrogen peroxide.

The proposed functions of NADPH oxidase-derived ROS in the kidney are mainly regulation of renal blood flow, alteration of cell fate, and regulation of gene expression. Superoxide avidly reacts with nitric oxide (NO) limiting its relaxing effect on afferent arterioles and mediates the activation of inflammasome, while hydrogen peroxide is involved in the activation of protein tyrosine kinases, phospholipases, serine/threonine kinases, and so forth, resulting in enhanced epithelial-to-mesenchymal transition (EMT), apoptosis of podocytes, and promotion of cellular hypertrophy [18–25].

The present review will focus on the role of NADPH oxidase-induced oxidative stress in the pathogenesis of metabolic disease-related renal injury.

## 2. NADPH Oxidase and Diabetic Nephropathy

Diabetic nephropathy (DN) is the major complication of type 1 and type 2 diabetes and is one of the leading causes of end-stage renal disease (ESRD) [26]. It is characterized by functional deficits with proteinuria and decreased glomerular filtration, as well as structural changes, such as loss of podocytes, proliferation and expansion of mesangial cells and matrix, thickening of glomerular and tubular basement membranes, tubular atrophy, interstitial fibrosis, and arteriosclerosis. Increasing evidence has demonstrated that NADPH oxidase-induced oxidative stress plays a pivotal role in the initiation and development of DN [11, 27]. Blockade of NADPH oxidase-derived ROS generation ameliorates diabetes-induced glomerular injury via reducing podocyte loss, proteinuria, glomerular hypertrophy, and mesangial matrix expansion [28–33].

Damage and depletion of podocytes due to apoptosis occur during early DN, presented as actin cytoskeleton rearrangement, podocyte foot process effacement, and slit diaphragm disruption [34]. Studies have highlighted the role of podocytes in DN pathogenesis and revealed the upregulation of the NADPH oxidase subunits expression, predominantly Nox4 and Nox1, in type 1 diabetic OVE26 mice and type 2 diabetic db/db mice, following excessive ROS generation and podocytes apoptosis which contributes to albuminuria [20, 35–37]. In vitro studies also have shown that high glucose induced the upregulation of NADPH oxidase expression, enhancement of NADPH oxidase activity, and apoptosis induction in podocytes at later time points [35, 37–39]. Eid et al. found that the increase of Nox4 expression was attributed to the inactivation of AMP-activated protein kinase (AMPK), and Nox4 promoted podocyte apoptosis via p53- and PUMA-dependent apoptotic pathway in high glucose condition [35, 40]. Other NADPH oxidase subunits, such as Nox2, p22phox, and p67phox, are also expressed on podocytes. However, very little is known concerning the regulation of these subunits in the presence of high glucose [11, 41–43].

Besides podocyte injury, two other morphological alterations during early DN are mesangial matrix accumulation and cell hypertrophy leading to thickening of glomerular basement membrane [27, 44]. The important role of NADPH oxidase in mesangial cell injury has been demonstrated in experimental models of diabetes as well as in cultured cells exposed to high glucose, while the molecular mechanisms remain speculative. High glucose induces upregulation of Nox4 and p22phox expression in mesangial cells as well as in diabetic kidney, and Nox4 and p22phox mediate cell hypertrophy and fibronectin expression [12, 45–48]. Since p22phox interacts with Nox4 and enhances its activity, Gorin and Wauquier suggested that p22phox and Nox4 might form a complex that contributed to high glucose-dependent oxidative stress and the subsequent fibrotic processes [13]. The role of other NADPH oxidase subunits in mesangial cell injury has been less studied and the findings are controversial.

Furthermore, NADPH oxidase also mediates the ROS generation induced by other mediators in DN such as Ang II and TGF-*β* [42, 49–51]. Induced by Ang II, an acute increase and prolonged upregulation of Nox4 expression both take place in mesangial cells, and Nox4 mediates ROS generation leading to activation of signalling, for instance, extracellular signal-regulated kinase-1/2 (ERK1/2) [52], Akt/protein kinase B (Akt/PKB) [50], and proline-rich tyrosine kinase-2 (Pyk-2)/Src/3-phosphoinositide-dependent protein kinase-1 (PDK-1) [22], which results in hypertrophy and increased fibronectin expression. Induced by TGF-*β*, Nox4 expression within mitochondria in podocytes is upregulated via the Sma and Mad homologue (Smad) 2/3 pathway and ultimately results in ROS overproduction, mitochondrial dysfunction, and podocyte apoptosis [53, 54].

## 3. NADPH Oxidase and Hyperhomocysteinemia-Associated Glomerular Injury

Hyperhomocysteinemia (hHcys) is defined as a pathological condition characterized by abnormal elevation of homocysteine (Hcys) plasma concentration and has been considered as a pivotal independent risk factor in the development of progressive glomerulosclerosis and/or ESRD [55–57]. Previous evidence has revealed that Hcys induces endothelial injury, vascular smooth muscle cells proliferation, and extracellular matrix (ECM) metabolism disturbance [58–61]. Considering the similarity of pathological alterations between Hcys-induced arterial injury and glomerular injury, the role of hHcys in glomerulosclerosis has been verified. Although the mechanism by which Hcys induces glomerular injury remains poorly understood, there is evidence that NADPH oxidase-derived oxidative stress is involved in the development of glomerular injury induced by Hcys [62–65]. An experimental model of hHcys was reported to develop glomerulosclerosis, characterized by local oxidative stress, podocyte dysfunction, mesangial expansion, and fibrosis, which could be significantly attenuated by treatment of NADPH oxidase inhibitors [64].

Podocyte injury is a critical early event leading to glomerulosclerosis. It has been revealed that Hcys induces podocyte damage and slit diaphragm disruption, causing proteinuria and glomerular sclerosis [66]. Zhang et al. [67] found that, in mice lacking Nox2 gene, hHcys induced by folate-free diet led to less severe podocyte injury and glomerulosclerosis, as shown by attenuated foot process effacement and podocyte loss, lower proteinuria, and glomerular damage index, as well as higher glomerular filtration rate. Thus, NADPH oxidase is suggested to be essential for Hcys-induced podocyte injury and glomerulosclerosis. Furthermore, Hcys stimulation was documented to upregulate NOX2 and p47phox expression and induce their aggregation in lipid raft (LR) clusters in podocytes, while disrupting LR clustering markedly blocked the enrichment of the NADPH oxidase subunits, decreased the enzyme activity, and functionally attenuated Hcys-induced podocyte injury. These findings indicate that NADPH oxidase subunits aggregation and activation through LR clustering are important molecular mechanisms in Hcys-induced podocytes injury [68]. Hcys is also confirmed to induce podocytes to undergo EMT and inflammasome activation through NADPH oxidase-derived oxidative stress, which consequently leads to glomerular injury and sclerosis [69–71].

Ingram's research group and others also have clarified that Hcys induces alterations of ECM metabolism in mesangial cells, another important event leading to glomerulosclerosis and loss of renal function [72]. Hcys was reported to upregulate tissue inhibitor of metalloproteinase-1 and induce collagen type I accumulation, accompanied by enhanced cell proliferation and NADPH oxidase activity in rat mesangial cells [73]. Hcys-induced activation of NADPH oxidase is suggested to be mediated by enhanced ceramide synthesis and the subsequent increase of Rac GTPase activity [74]. There is also evidence showing that the N-methyl-D-aspartate (NMDA) receptor may mediate activation of NADPH oxidase in hHcys-associated glomerular injury [75]. In addition, Hcys has been found to cause mesangial apoptosis via oxidative stress and p38-mitogen-activated protein kinase activation, thereby suggesting another underlying mechanism of hHcys-associated glomerular injury [63].

## 4. NADPH Oxidase and Hyperlipidemia-Associated Glomerular Injury

The concern of the association between hyperlipidemia and renal diseases may date back to the 19th century. Since then, accumulating evidence in experimental findings and clinical observations has suggested an important role of hyperlipidemia in the progression of glomerulosclerosis [76–81]. Hyperlipidemia-associated glomerular injury is mainly characterized by lipid or lipoprotein deposition, macrophage infiltration, and mesangial expansion. As with other metabolic factors, such as hyperglycemia and hHcys, oxidative stress is proved to contribute to the deleterious effects of hyperlipidemia on renal injury. In high-fat diet fed mice, the expression of NADPH oxidase subunits, including p47phox, Nox2, and p67phox, was significantly upregulated, and the inhibitor could ameliorate hyperlipidemia-induced endothelial dysfunction via inhibition of NADPH oxidase expression [82]. However, in the study of Scheuer et al., it is reported that xanthine oxidoreductase rather than NADPH oxidase mainly accounted for the generation of ROS in glomeruli and tubulointerstitium induced by hyperlipidemia [83]. In addition, Joles et al. clarified that both hypercholesterolemia and hypertriglyceridemia aggravated renal injury predominantly via podocytes, accompanied by activation and injury of tubulointerstitial cells, lacking evidence of mesangial activation, proliferation, or matrix accumulation [80]. Furthermore, hyperlipidemia often coexists with other metabolic syndrome components and accelerates the progression of glomerular injury together [84, 85].

## 5. NADPH Oxidase and Hyperuricemia-Related Kidney Disease

Uric acid (UA) is an intermediate product in the purine degradation pathway in cells but is the final product of purine catabolism in humans, due to the loss of uricase activity during hominoid evolution [86]. The role of UA in CKD remains controversial, and the “UA debate” has been going on for decades [87]. UA has been considered as a major antioxidant in protecting cells from oxidative injury proved by abundant experimental and clinical evidence [88]. On the other hand, epidemiologic evidence and experimental models also have shown that hyperuricemia may impose detrimental effects as a prooxidant [89–92]. UA is often associated with other risk factors of CKD, including diabetes, hypertension, and inflammation [93], which makes it difficult to dissect the role of UA itself in the progression of CKD. However, a recent study showed an association between hyperuricemia and renal damage independently of hypertension and intrarenal renin-angiotensin system (RAS) activation [94].

In the past, hyperuricemia was thought to cause kidney disease by a crystal-dependent mechanism. The crystal of monosodium urate may induce potassium efflux, lysosomal rupture, and mitochondrial ROS production, which provoke inflammasome and induce the secretion of proinflammatory cytokines, eventually causing inflammation and renal injury. The crystal-independent mechanism of hyperuricemia-related kidney disease remains poorly understood. The main pathophysiological mechanisms of hyperuricemia-related kidney disease include endothelial dysfunction, activation of local RAS, oxidative stress, and proinflammatory and proliferative effects. NADPH oxidase is suggested to play a role in the pathogenesis of hyperuricemia-related kidney disease, as with other metabolic disease-related renal injuries. It has been revealed that hyperuricemia is associated with endothelial dysfunction, due to oxidative stress with activation of RAS and a decrease of NO bioavailability [95, 96]. In an experimental model of hyperuricemia, enhanced intrarenal oxidative stress, increased expression of NOX-4 and Ang II, and decreased NO bioavailability were observed [97]. The aging and apoptosis of endothelial cells induced by hyperuricemia were ameliorated by antioxidants [98]. Furthermore, there is evidence that mitochondrial alterations and decreased intracellular ATP are implicated in UA-induced endothelial dysfunction [99]. In cultured renal tubular cells, it has been shown that UA induces EMT and apoptosis of renal tubular cells which is ameliorated by antioxidants, suggesting a detrimental role of oxidative stress [100].

## 6. NADPH Oxidase and Obesity-Related Kidney Disease

Oxidative stress is also associated with other metabolic kidney diseases such as obesity-related kidney disease [101]. It is well documented that the glomerular scarring in obesity-associated focal segmental glomerulosclerosis is driven by podocyte injury, which may partly be a result of the NADPH oxidase-derived oxidative stress induced by upregulated Ang II and TGF-*β* [102]. There is supplemental data supporting the fact that NADPH oxidase-mediated oxidative injury to the proximal tubule contributes to proteinuria in obese rats [103]. In addition, oxidative stress is demonstrated to play a role in the pathogenesis of renal injury through its contribution to progressive vascular dysfunction and remodeling [104, 105]. Collectively, NADPH oxidase-derived oxidative stress is suggested to trigger the progression of obesity-related kidney disease.

## 7. Conclusion

**Figure 1:**
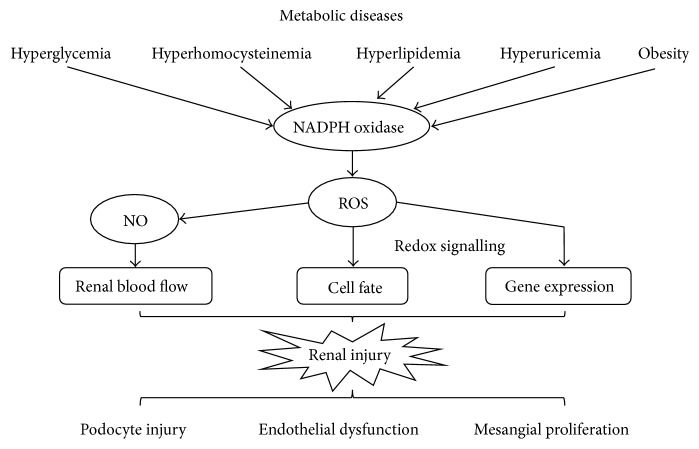
NADPH oxidase-derived ROS in the pathogenesis of metabolic disease-related renal injury. Metabolic stimuli may upregulate the expression of NADPH oxidase and enhance the activity of NADPH oxidase, which subsequently leads to overproduction of ROS. NADPH oxidase-derived oxidative stress is involved in podocyte injury, endothelial dysfunction, mesangial proliferation, and so forth, eventually resulting in renal injury. NADPH: nicotinamide adenine dinucleotide phosphate; ROS: reactive oxygen species; NO: nitric oxide.

The NADPH oxidase is widely expressed throughout the kidney and is a major source of intrarenal oxidative stress. Metabolic stimuli elicit the upregulation of NADPH oxidase expression and the enhancement of NADPH oxidase activity. As depicted in Figure 1, ROS generated by NADPH oxidase plays a pivotal role in the pathogenesis of glomerular diseases related to metabolic diseases. Hence, approaches to reduce oxidative stress by antioxidants may be potential therapies to prevent and treat metabolic disease-related renal injury.
